# The role of nitric-oxide-synthase-derived nitric oxide in multicellular traits of Bacillus subtilis 3610: biofilm formation, swarming, and dispersal

**DOI:** 10.1186/1471-2180-11-111

**Published:** 2011-05-20

**Authors:** Frank Schreiber, Martin Beutler, Dennis Enning, María Lamprecht-Grandio, Olga Zafra, José Eduardo González-Pastor, Dirk de Beer

**Affiliations:** 1 Max-Planck-Institute for Marine Microbiology, Celsiusstrasse 1, D-28359 Bremen, Germany; 2 bionsys GmbH, Fahrenheitstrasse 1, D-28359, Bremen, Germany; 3 Instituto Nacional de Téchnica Aeroespecial, Centro de Astrobiologia (INTA-CSIC), Madrid 28850, Spain; 4 Eawag - Swiss Federal Institute of Aquatic Science and Technology, Überlandstrasse 133-P.O. Box 611-8600 Dübendorf-Switzerland

## Abstract

**Background:**

*Bacillus subtilis *3610 displays multicellular traits as it forms structurally complex biofilms and swarms on solid surfaces. In addition, *B. subtilis *encodes and expresses nitric oxide synthase (NOS), an enzyme that is known to enable NO-mediated intercellular signalling in multicellular eukaryotes. In this study, we tested the hypothesis that NOS-derived NO is involved in the coordination of multicellularity in *B. subtilis *3610.

**Results:**

We show that *B. subtilis *3610 produces intracellular NO *via *NOS activity by combining Confocal Laser Scanning Microscopy with the NO sensitive dye copper fluorescein (CuFL). We further investigated the influence of NOS-derived NO and exogenously supplied NO on the formation of biofilms, swarming motility and biofilm dispersal. These experiments showed that neither the suppression of NO formation with specific NOS inhibitors, NO scavengers or deletion of the *nos *gene, nor the exogenous addition of NO with NO donors affected (i) biofilm development, (ii) mature biofilm structure, and (iii) swarming motility in a qualitative and quantitative manner. In contrast, the *nos *knock-out and wild-type cells with inhibited NOS displayed strongly enhanced biofilm dispersal.

**Conclusion:**

The results suggest that biofilm formation and swarming motility in *B. subtilis *represent complex multicellular processes that do not employ NO signalling and are remarkably robust against interference of NO. Rather, the function of NOS-derived NO in *B. subtilis *might be specific for cytoprotection against oxidative stress as has been proposed earlier. The influence of NOS-derived NO on dispersal of *B. subtilis *from biofilms might be associated to its well-known function in coordinating the transition from oxic to anoxic conditions. Here, NOS-derived NO might be involved in fine-tuning the cellular decision-making between adaptation of the metabolism to (anoxic) conditions in the biofilm or dispersal from the biofilm.

## Background

Nitric oxide (NO) is a signalling molecule in multicellular, eukaryotic organisms, where it coordinates the function and interactions between cells of the cardiovascular, neuro, and immune system [1]. These cells have the ability to synthesize NO with the enzyme NO synthase (NOS) using arginine and O_2 _as substrates [2]. The targets of NO signalling are mainly NO-mediated protein modifications, such as iron-nitrosylation and S-nitrosylation of active site cysteine thiols. These modifications critically depend on the apparent NO concentration and the redox conditions. Thus, NO signalling is considered to be a redox-based signalling event [3].

Functional NOS was also found to be encoded and expressed in certain, predominately gram-positive, bacteria including the well-studied model organisms *Bacillus subtilis *[4,5]. Until now, only few studies reported on the function of NOS-derived NO in bacteria. Gusarov and Nudler [6] showed that NOS-derived NO in *B. subtilis *provides instant cytoprotection against oxidative stress imposed by H_2_O_2 _with two different mechanisms. Firstly, NO activates catalase, the H_2_O_2 _degrading enzyme. Secondly, NO suppresses cytotoxic Fenton chemistry - the formation of DNA damaging OH· radicals from the oxidation of Fe^2+ ^with H_2_O_2_. Here, NO interrupts the re-supply of Fe^2+ ^by inhibiting the enzymatic reduction of cysteine, which controls the (re-)reduction of intracellular Fe^3+ ^to Fe^2+^. This alleviation from oxidative stress by NOS-derived NO has been shown to be partly responsible to protect bacteria against a range of antibiotics that induce oxidative stress [7]. A completely different function of NOS-derived NO was described in *Streptomyces turgidiscabies*, where it is involved in the biosynthesis of a secondary metabolite (a dipeptide phytotoxin) by the site-specific nitration of a tryptophanyl moiety [8].

In addition, NO is an established signalling molecule in bacteria interacting with many bacterial regulatory components, such as OxyR, SoxR, NsrR, NorR and regulators of the FNR family [9]. In these systems, the NO signal is mainly thought to be produced as an intermediate or by-product of catabolic reactions of the nitrogen cycle or from eukaryotic host cells that attack pathogens with NO. However, the fact that certain bacteria encode and express NOS prompted the hypothesis that NOS-derived NO is involved in intercellular signalling between bacteria to exert multicellular functions [10].

Signalling in bacteria is especially important for the coordination of their multicellular traits. Remarkable multicellular traits in bacteria are swarming motility and biofilm formation, both of which have been intensively studied in *B. subtilis *NCIB3610 [11-15]. This strain was isolated ~1930 and is probably the progenitor of the sequenced laboratory strain *B. subtilis *168, which does not exhibit swarming motility and formation of structural complex biofilms, because it is thought to have lost these traits by intense laboratory use for decades (domestication) [11,16,17].

Swarming motility is a multicellular movement of bacteria that migrate above solid substrates in groups of tightly bound cells [18]. Swarming motility is dependent on cellular differentiation processes of sessile or swimming cells into swarm cells, which are longer, more flagellated and can assemble into multicellular rafts. The differentiation into swarm cells and the swarm expansion is thus a multicellular process that is governed by signals that coordinate the interaction between individual cells. *B. subtilis *displays many of the classical features of swarming motility. When centrally inoculated on nutrient-rich agar (0.5-0.7% agar) cells differentiate into swarm cells and, after a lag phase of a few hours, expand rapidly over the entire agar surface [13]. The swarm edge consists of poorly motile cells that are driven forward by motile, highly flagellated cells that are organized in multicellular rafts.

Biofilm formation in *B. subtilis *is characterized by the formation of robust pellicles at the air-liquid interface and the formation of structurally complex spot colonies on agar surfaces. Within biofilms *B. subtilis *forms aerial projections called fruiting bodies, because their tips are the preferential sites for sporulation [12]. A hallmark of biofilm development in *B. subtilis *is the differentiation of the *B. subtilis *population into different subpopulations. Phosphorylation of the master regulator Spo0A controls differentiation. The subpopulation with low intracellular levels of phosphorylated Spo0A produces the extracellular matrix, while the subpopulation with high intracellular levels of phosphorylated Spo0A differentiates into spores [14]. A set of specific sensor kinases (KinA, B, C, D, and E) controls the level of Spo0A phosphorylation, but the extra- or intracellular signals that affect these kinases remain largely unknown [14]. Signalling molecules for *B. subtilis *differentiation events that are known to date are mostly specific peptides, such as ComX, sufactin, and PhrC. In this study, we hypothesized that biofilm formation in *B. subtilis *is controlled by the redox-based signal of NOS-derived NO, in addition to a response to structurally specific signalling molecules.

Another important aspect of biofilm physiology is the dispersal of cells from the biofilm. Biofilm dispersal is defined as a process in which initially sessile cells undergo phenotypic modifications, which enable them to actively leave the biofilm and finally convert to planktonic cells [19,20]. Active biofilm dispersal contrasts the process of passive sloughing of cells from the biofilm by hydrodynamic stress. *Pseudomonas aeruginosa *is an important model system for studying biofilm dispersal. Here, previous studies have shown that dispersal can be considered a multicellular trait as it involves quorum sensing [21]. However, the underpinnings of biofilm dispersal are the metabolic state of the biofilm cells, as regulatory systems for dispersal are controlled by nutrient availability [22-24]. Dispersal of *B. subtilis *biofilms has not been investigated to date even though its apparent fruiting bodies have led to the speculation about their function in spore dispersal [12].

In this study we hypothesized that NOS-derived NO coordinates multicellular traits of *B. subtilis *3610. We examined the effect of exogenously supplied NO and of NOS inactivation on biofilm formation, swarming motility and biofilm dispersal in *B. subtilis*. The results show that NOS and NO do not affect biofilm formation and swarming, but unambiguously show an influence of NOS on biofilm dispersal.

## Results and Discussion

### NO formation in *B. subtilis *3610

**Figure 1:**
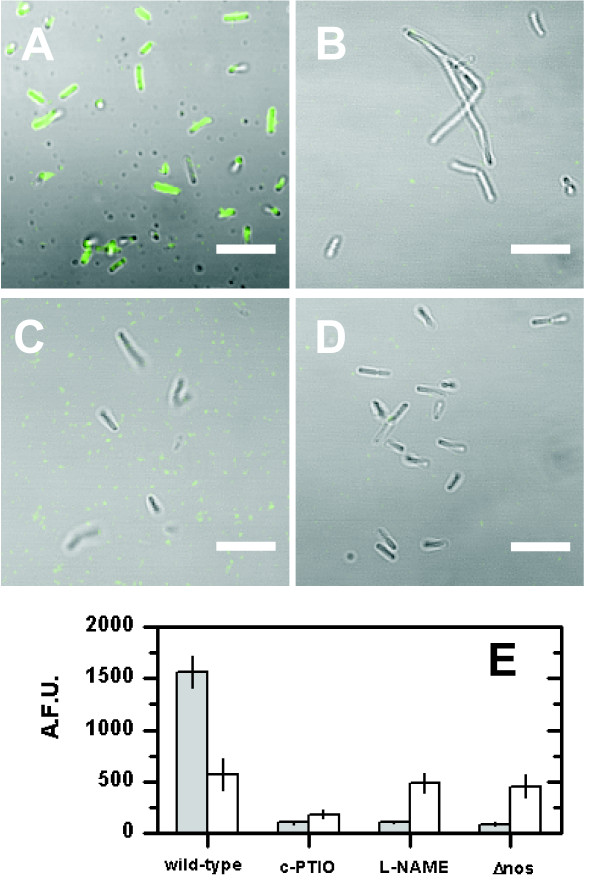
**Nitric-oxide-synthase (NOS)- derived NO formation by *B. subtilis *3610**. (**A-D**) Confocal laser scanning micrographs of cells grown in LB for 4 h at 37°C. Shown is the overlay of: gray - transmission and green - fluorescence of NO sensitive dye CuFL. **(A) **Wild-type without supplements, **(B) **supplemented with 100 μM c-PTIO (NO scavenger), **(C) **100 μM L-NAME (NOS inhibitor), and **(D) **3610Δ*nos*. Scale bar is 5 μm. **(E) **Single-cell quantification of intracellular NO formation of cells grown in LB (gray bars) and MSgg (white bars) using CuFL fluorescence intensity (A.F.U. = Arbitrary Fluorescence Units). Error bars show standard error (N = 5).

We tested intracellular production of NO in *B. subtilis *3610 grown in LB and in MSgg medium by staining cells with the NO sensitive dye CuFL. The results show that wild-type *B. subtilis *produces NO in both media (Figure 1). Incubation of wild-type cells with the NO scavenger c-PTIO decreased NO production to 7% in LB and 33% in MSgg as compared to untreated wild-type cells (Figure 1A, B &1E). This confirms that NO signals measured in the untreated wild-type are specific to NO and were not derived by unspecific reactions of the dye with other cellular components. Incubation of wild-type cells in LB with the NO synthase (NOS) inhibitor L-NAME and of a mutant that lacked the *nos *gene decreased in both cases NO production to ~ 7% as compared to untreated wild-type cells (Figure 1C-E). In contrast, supplementing MSgg medium with the NOS inhibitor L-NAME and growing the *nos *mutant in MSgg decreased NO production to only 85% and 80%, respectively, as compared to untreated wild-type cells (Figure 1E).

The data shows that *B. subtilis *uses NOS to produce NO in LB and indicates that NO production *via *NOS is low in MSgg. Furthermore, the NO scavenger c-PTIO effectively reduces intracellular NO and the NOS inhibitor L-NAME inhibits NO formation by NOS. Hence, both compounds are suitable tools to test the effect of NO and NOS-derived NO, respectively, on multicellular traits of *B. subtilis*. Moreover, the data indicates that *B. subtilis *produces significant amounts of NO with an alternative mechanism besides NOS when grown in MSgg. An alternative pathway of NO formation in *B. subtilis *could be the formation of NO as a by-product during the reduction of NO_2_^- ^to ammonium (NH_4_^+^) by the NO_2_^- ^reductase NasDE [25]. Both LB (~35 μM) and MSgg (~ 5 μM) contained traces of oxidized inorganic nitrogen (NO_3_^- ^or NO_2_^-^; NO_x_), which might be a sufficient source for low nanomolar concentrations of NO even if most NO_x _is reduced to NH_4_^+^. Gusarov et al. [26] showed that NasDE actively reduces NO_x _in LB-cultures at the end of the stationary phase. However, NO production from ammonifying NO_2_^- ^reductases has so far only been reported for the ammonifying NO_2_^- ^-reductase Nrf of *E. coli *[27], but not for NasDE of *B. subtilis*. The potential ability of NasDE to generate NO may be an interesting subject for further research directed toward the understanding of how *B. subtilis *controls NO homeostasis under different environmental conditions.

### NO is not involved in biofilm formation of *B. subtilis *3610

**Figure 2:**
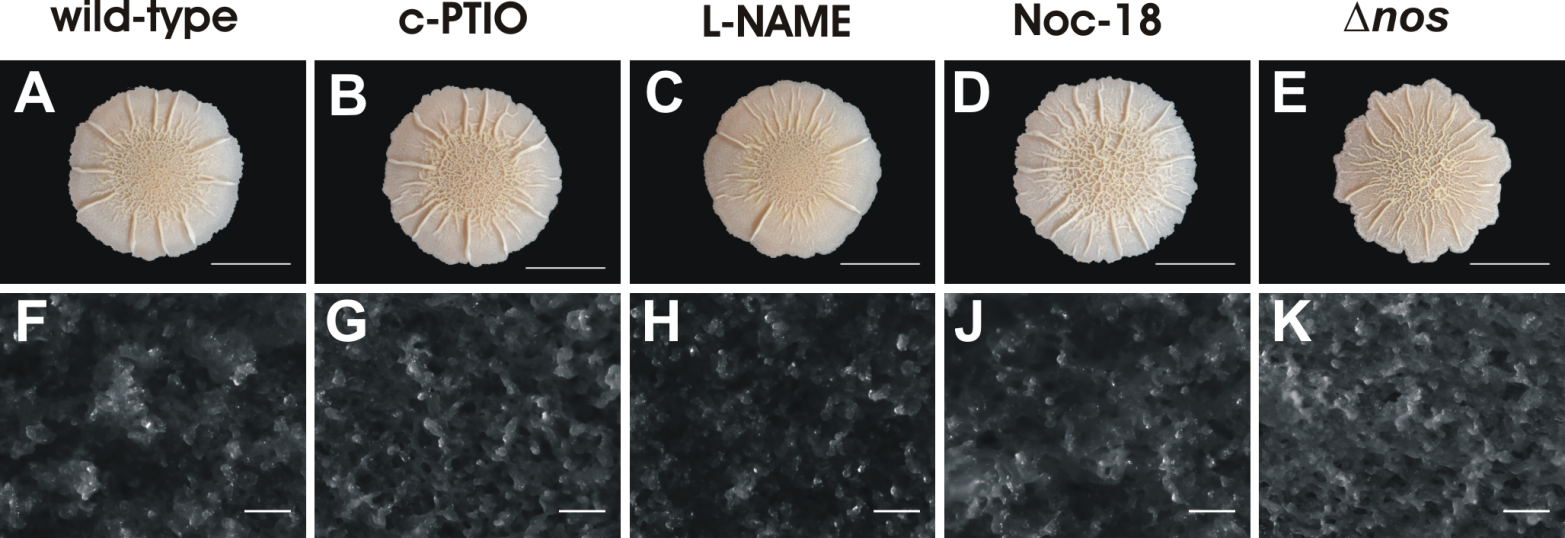
**Influence of NO and NO synthase (NOS) on colony morphology and fruiting body formation of *B. subtilis *3610**. **(A-E) **Colonies were grown for 4 d on MSgg agar and images were captured with a digital camera. **(F-K) **Colonies were grown for 3 d on MSgg agar and images were captured with a CCD camera mounted on a microscope. NO scavenger (c-PTIO), NOS inhibitor (L-NAME) and NO donor (Noc-18) were added to biofilm incubations of *B. subtilis *wild-type. Scale bars are 1 cm **(A-E) **and 200 μm **(F-K)**.

We tested the influence of NOS-derived NO and exogenously supplemented NO on biofilm formation of *B. subtilis *3610 by monitoring the morphology of agar-grown colonies and the development of biofilms on the air-liquid interface (pellicles) in MSgg medium. The results show that the development of structured colonies on agar surfaces is not influenced by an NOS inhibitor, an NO scavenger, the addition of NO with an NO donor and by deleting the gene that encodes for NOS (Figure 2A-E). Furthermore, the treatments did not affect the development of structures described earlier as fruiting bodies [12] in the colony biofilms (Figure 2F-K). In addition, we monitored the developmental sequence of pellicle formation on the cellular level with phase contrast microscopy (data not shown). Pellicles developed regardless of the treatment from motile cells of unit length, over non-motile cells aligned in long chains, to densely packed cells and spores, which resemble the developmental sequence described by Branda et al. 2001 [12].

**Figure 3:**
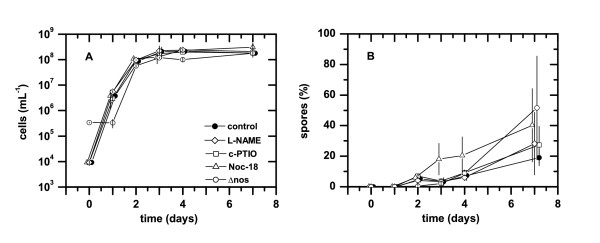
**Influence of NO and NO synthase **(A) **on the cell concentration and **(B) **the percentage of spores per cell during the development of biofilms of *B. subtilis *3610 and 3610Δ*nos *at the liquid-air interface as determined by plate counting**. Biofilms of wild-type 3610 were grown in 25 mL MSgg medium in glass tubes without supplementation (control), supplemented with 100 μM L-NAME (NOS inhibitor), 75 μM c-PTIO (NO scavenger), and 130 μM Noc-18 (NO donor). Error bars indicate standard deviation (N = 3).

The quantitative growth kinetics of vegetative cells in the pellicle biofilms was not affected by the presence of NOS inhibitor, NO scavenger, NO donor, and a mutation in the *nos *gene (Figure 3A). Spore counts in the pellicles showed that the presence of NOS inhibitor and NO scavenger did not change the kinetics of spore formation (Figure 3B). In contrast, the presence of NO donor approximately doubled the number of spores in the early stages (day 3 and 4) of pellicle formation (Figure 3B). Measurements with NO and O_2 _microelectrodes showed that the addition of NO donor led to ~20 μM NO after 3-4 d of incubation in the anoxic medium underlying the pellicle, while NO could not be detected in the other treatments. The high NO concentration can exert toxic effects on the cells and might enhance spore formation. However, the structural assembly of spores in the biofilm was not affected (data not shown) and the differences in spores were not significant between treatments in the mature biofilms after 7 days of incubation.

Intracellular measurements of NO in *B. subtilis *indicated that NO production from NOS is low in MSgg medium (Figure 1E), which is typically used to induce formation of structurally complex *B. subtilis *biofilms [14]. This might explain the absence of an effect of NOS inhibitors and *nos *knock-out on biofilm formation. In addition, biofilm formation is not affected by NO produced by other NO-producing pathways, as neither the NO scavenger nor the addition of exogenous NO had an effect on mature biofilm structures.

Previous studies have shown that cellular differentiation and biofilm formation in *B. subtilis *are controlled by intracellular concentrations of the phosphorylated master regulator Spo0A [14]. Two sensor kinases (KinA and KinC) that control the level of Spo0A phospohrylation possess cytoplasmic PAS sensor domains, which have been implicated to sense redox potential and O_2_. In turn, a mutational study of the cytoplasmic PAS domain of *B. subtilis' *sensor kinase ResE suggested that it senses NO under anaerobic conditions [28]. Thus, it is conceivable that KinA and KinC are affected by NO signalling. However, our study indicates that NOS-derived NO and exogenously supplied NO do not affect the PAS domains of KinA and KinC such that biofilm formation and differentiation is significantly altered. This supports the notion that biofilm formation and differentiation in *B. subtilis *are rather controlled by specific extracellular molecules, such as signalling peptides [14], as opposed to more broad range redox-based signals like NO.

### NO is not involved in coordinating swarming of *B. subtilis *3610

**Figure 4:**
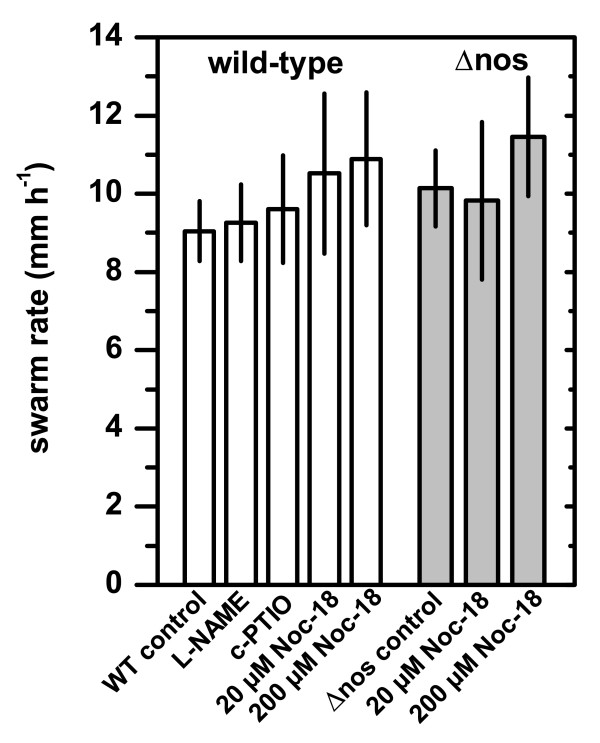
**Influence of NO and NO synthase (NOS) on the swarm rate of *B. subtilis *3610**. Swarm expansion assays with strain 3610 wild-type (white bars) and strain 3610Δ*nos *(gray bars) were performed on 0.7% LB agar without supplementation (controls) or supplemented with 100 μM L-NAME (NOS inhibitor), 100 μM c-PTIO (NO scavenger) and 20 μM or 200 μM Noc-18 (NO donor). Error bars indicate standard deviation (N = 6). Differences between individual dataset are not statistically significant (α = 0.01; see Material and Methods section for details).

We tested the influence of NO and NOS activity on the swarming motility of *B. subtilis *3610 on LB-based swarm agar (Figure 4). Swarm expansion of wild-type *B. subtilis *on 0.7% LB agar was 9 mm h^-1 ^(± 0.8 mm) and agrees well with swarm expansion of 10 - 14 mm h^-1 ^reported by Kearns and Losick [13]. Swarm expansion was not significantly affected by the presence of NOS inhibitors, NO scavenger, NO donor and for the *nos *mutant. This shows that neither NOS-derived NO nor exogenously supplied NO influences swarming motility in *B. subtilis*.

### NOS-derived NO inhibits biofilm dispersal of *B. subtilis *3610

**Figure 5:**
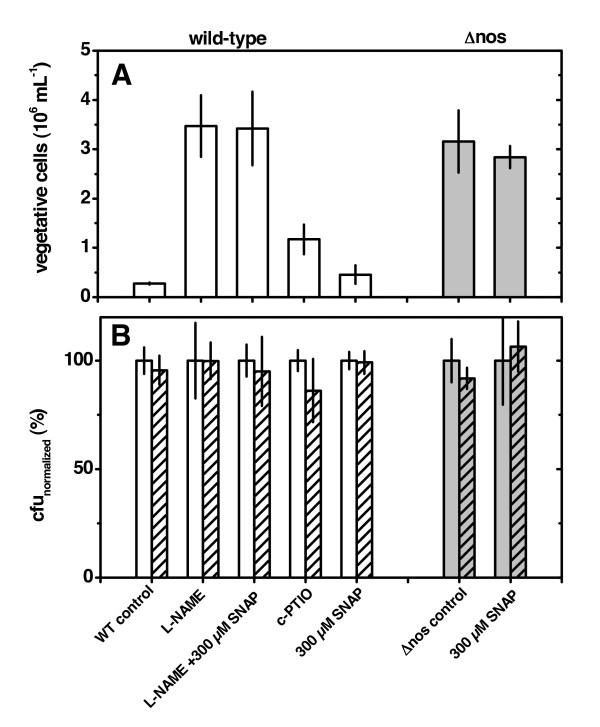
**Influence of NO and NO synthase on **(A) **dispersal and **(B) **germination of *B. subtilis *3610**. **(A) **The dispersal assay was conducted with 3610 wild-type (white bars) and 3610Δ*nos *(gray bars). Colonies grew for 4 d on MSgg agar and were mounted with a drop of 100 μL MSgg medium. The NOS inhibitor L-NAME and the NO scavenger c-PTIO were supplemented to agar and drop, while the NO donor SNAP was only supplemented to the drop. Vegetative cells that dispersed within 2 h into the drop liquid were quantified with flow cytometry. Error bars indicate standard error (N = 10). **(B) **The germination assay was conducted in a separate experiment, employing a similar set-up and the same treatments as for the dispersal assay. MSgg medium (including supplements) was mixed with *B. subtilis *spores, placed as a 100 μL drop on a sterile polystyrene surface and incubated for 2 h. Spores only (open bars in panel B) and total cells (hatched bars in panel B) were determined by plating and counting the colony forming units (cfu). The results are normalized to the spore concentration. Error bars indicate standard deviation (N = 5). The results show that any difference in the dispersal assay is caused by effects of NO and NOS on active dispersal of vegetative biofilm cells and not on germination of spores.

We tested the influence of NOS-derived NO and exogenously supplied NO on the dispersal of *B. subtilis *3610 from spot colony biofilms of wild-type and *nos *mutant cells (Figure 5A). First, biofilms were grown on MSgg agar or MSgg agar supplemented with NOS inhibitor or NO scavenger. To assay dispersal, we mounted a drop of MSgg medium containing a similar treatment as the underlying agar onto mature colony biofilms. The cell number in the drop was determined after short-term incubation (2 h) to minimize growth in the drop. While it is not expected that considerable growth occurs, any minor growth will proceed with a similar rate in all treatments (Figure 3A). In addition, placing the drop on the biofilm may cause some cells to enter the liquid by mechanical forces. However, those will be similar in all treatments and in the control that is done with MSgg only. Thus, differences in cell number in the drop entirely reflect differences in active dispersal of cells from the biofilm into the drop. Using flow cytometry we distinguished vegetative cells and spores, which presumably have no means of active dispersal as they are in an inactive state.

The results showed that dispersal is ~10 fold enhanced in the *nos *mutant and when the wild-type strain is subjected to NOS inhibitors (Figure 5A). Additionally, the presence of the NO scavenger c-PTIO increased the dispersal 4 fold. These results suggest that NOS is involved in a mechanism that facilitates the maintenance of cells in the biofilm. The fact that both NOS inhibitor and *nos *deletion increased dispersal argues against an unspecific effect of the deletion of the *nos *gene on dispersal.

The amount of vegetative cells present in the drop would increase if inhibition of NO synthesis increases the germination rate, because spores that are abundant in the tips of the fruiting bodies would germinate faster and release more vegetative cells. To exclude this possibility we measured germination of spores - derived from a defined spore solution - inside an MSgg drop without underlying biofilm. The results show that germination does not occur during 2 h incubation time in MSgg and that neither NOS inhibition nor deletion of the *nos *gene accelerated germination (Figure 5B). This confirms that any difference in the dispersal assay is caused by effects of NO and NOS on active dispersal of vegetative biofilm cells and not on germination of spores.

**Figure 6:**
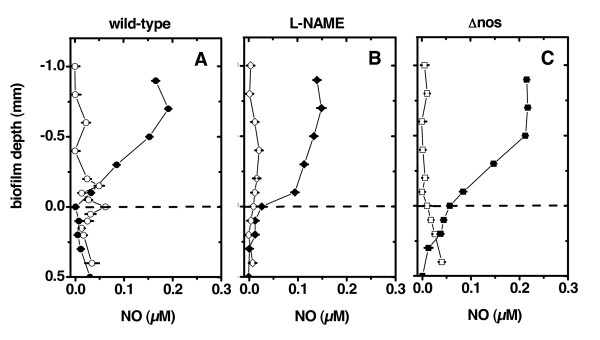
**Nitric oxide microprofiles measured during the dispersal assay**. The y-axis shows the biofilm depth with 0 (dashed line) denoting the surface of the biofilm. Positive values are inside the spot colony biofilm and negative values are above the biofilm in the MSgg medium drop. MSgg medium was supplemented with 300 μM of the NO donor SNAP (closed symbols) or supplied without supplementation of SNAP (open symbols). Wild-type *B. subtilis *3610 was incubated with a drop of MSgg **(A) **without further supplementation and **(B) **further supplemented with 100 μM NOS inhibitor L-NAME. (**C) **shows *B. subtilis *3610 Δ*nos *supplied with MSgg without further supplementation. Error bars depict the standard deviation (N = 10) between repeated measurements at the same position in the sample reflecting the precision of the measurement.

Interestingly, the addition of exogenously supplied NO with the chemical NO donor SNAP to the *nos *mutant and L-NAME-inhibited wild-type cells did not restore dispersal to wild-type levels. We used NO microsensors to measure whether the extracellular NO concentrations established by the NO donor during the dispersal assay were sufficient to complement for the loss of NOS synthesis. We found that addition of 300 μM SNAP to the dispersal drop resulted in an NO concentration between 150 to 200 nM (Figure 6). NO was consumed within the biofilm resulting in NO concentrations around the lower detection limit (~ 30 nM). Apparent NO consumption did not depend on the ability of *B. subtilis *to synthesize NO with NOS. NO concentrations within biofilms not exposed to the NO donor were also around the lower detection limit and could not be quantified with confidence. Thus, we could not discern if similar extracellular concentrations of NO were present during the different treatments in the biofilm microenvironment.

Taken together the results show that the addition of the NO donor during the dispersal experiment potentially provided a sufficient flux of extracellular NO to complement the deficiency for NO synthesis. The apparent failure of complementation suggests that NOS-derived NO is not an *intercellular *signalling molecule for the maintenance of cells in the biofilm. Rather, it mediates its effect on dispersal at defined *intracellular *concentrations, which cannot be restored by the exogenous addition of NO. Defined intracellular NO concentrations would be particularly important if NOS-mediated signalling proceeds via redox-based modifications of enzymes [3] or when it is used for biosynthesis of other signalling molecules [8]. Our results suggest that one of these two mechanisms might act within *B. subtilis *cells to facilitate the maintenance of cells in the biofilm.

Kolodkin-Gal et al. [29] described the disassembly of *B. subtilis *biofilms triggered by self-produced D-amino acids. In this study, disassembly was characterized by a complete breakdown of the macroscopic biofilm structure upon accumulation or experimental addition of certain D-amino acids, because their insertion into the cell wall disrupted the bonding between cells and the extracellular matrix protein TasA. Generally, active dispersal of cells from biofilms does not necessarily involve complete biofilm disassembly, which might be viewed as an extreme case of dispersal. Thus, it is likely that other NOS-affected mechanisms exist that enable biofilm-residing *B. subtilis *to disperse without disrupting the entire biofilm structure.

The results are in contrast to earlier observation with *P. aeruginosa *and other bacteria which showed that exogenous addition of non-toxic NO concentrations led to a marked dispersal of biofilms that grew adhered to a solid surface [30-32]. This suggests that the effect of NO on dispersal is a species-specific phenomenon with different bacteria using NO for opposing dispersal strategies. Thus, NO and NOS inhibitors might be used in medical or technological applications to selectively induce dispersal of certain (undesired or pathogenic) bacterial groups in multi-species biofilms, while other (desired or harmless) bacteria may be selectively maintained in the biofilm. Alternatively, the different effects of NO on dispersal might be explained by the different types of dispersal assays and NO donors used in our study as compared to the study with *P. aeroginosa *[30].

Well-known bacterial regulatory systems that respond to NO as a signal are commonly associated to the onset of anaerobic respiration of NO_x _during the transition form oxic to anoxic conditions [9,33]. Also dispersal from biofilms can be considered a response to anoxia considering that a significant part of the biofilm cells resides in the anoxic layer of a biofilm. This might explain *why *the transition from aerobic to anaerobic metabolism and biofilm dispersal are both affected by NO signalling. For example, NO produced by denitrification in *P. aeruginosa *biofilms has been shown to control expression of denitrification genes [33,34] and to mediate dispersal [30]. Comparably, in *B. subtilis *it is already known that NO regulates the expression of *nasD *and *hmp*, a NO_2_^- ^- reductase and an NO detoxifying enzyme, respectively [35,36], while our findings link NOS-derived NO to dispersal of *B. subtilis*. The specific function of NOS in this context might be fine-tuning the cellular decision for either onset of anaerobic respiration or dispersal form the biofilm.

### NO connections between bacterial and metazoan multicellularity?

Numerous enzymes and regulators are involved in biofilm formation and swarming of *B. subtilis*. From our data it can be concluded that these traits of *B. subtilis *are remarkably stable against NO-mediated protein modifications, such as iron-nitrosylation and S-nitrosylation of cysteine thiols. Interpreted from an ecological perspective, multiple targets of NO signalling might be too unspecific for mediating specific cellular differentiation events, because in nature *B. subtilis *and other bacteria typically exist in multi-species biofilm communities. In other words, bacterial signalling systems that coordinate specific cellular differentiation events might be stable against NO modifications to avoid detrimental cross-talk with NO production by community members. Likewise, we showed that *B. subtilis *consumes exogenously supplied NO, maintaining extracellular NO levels (Figure 6). Specificity of NO as an intercellular signal for coordination of multicellular functions might have evolved in multicellular eukaryotes with the onset of the immune system, which guaranteed predictable sender and receivers of NO signals. This major evolutionary transition might have rendered certain proteins sensitive to NO-mediated post-translational modifications to use the wide potentials of redox-based NO signalling.

## Conclusions

This study shows that bacterial NOS is not involved in developmental processes and coordination of multicellular traits that are essential for biofilm formation and swarming motility in *B. subtilis*. NOS activity affects biofilm dispersal of *B. subtilis *warranting further investigations toward NOS-derived NO as an important mediator of bacterial responses to changing environmental and metabolic conditions. Moreover, our study supports the specificity of NOS-derived NO in cytoprotection against oxidative stress [6] and for specific nitration reactions biosynthetic pathways [8].

## Methods

### Bacterial strains, media and growth conditions

The experiments were conducted with *Bacillus subtilis *NCIB3610 obtained from Bacillus genetic stock center (Ohio State University, Columbus OH). Frozen glycerol (15%) stocks were revived overnight at 37°C on a rotary shaker in 50 mL Luria-Bertani (LB) broth, containing 10 g L^-1 ^tryptone, 5 g L^-1 ^yeast extract, 5 g L^-1 ^NaCl. For experiments, 1 mL of the overnight culture was freshly inoculated into 50 mL LB and cells were harvested in the mid-exponential phase after ~4-5 h of growth. LB medium fortified with 0.7% agar was used in swarm expansion assays. Minimal salt glycerol glutamate (MSgg) medium was prepared according to Branda et al. [12] containing 5 mM potassium phosphate (pH 7), 100 mM MOPS (pH 7), 0.5% glycerol, 0.5% glutamate, 50 mg L^-1 ^tryptophan, 50 mg L^-1 ^phenylalanine, 2 mM MgCl_2_, 0.7 mM CaCl_2_, 50 μM MnCl_2_, 50 μM FeCl_3_, 1 μM ZnCl_2_, 2 μM thiamine. Except for the swarming assay, experiments with 3610Δ*nos *were performed in media supplemented with 1 mg L^-1 ^erythromycin and 25 mg L^-1 ^lyncomycin.

The influence of NO on wild-type *B. subtilis *was tested with supplementation of NOS inhibitor N_ω_-Nitro-L-arginine methyl ester hydrochloride (L-NAME), NO scavenger 2-(4-Carboxyphenyl)-4,4,5,5-tetramethylimidazoline-1-oxyl-3-oxide potassium salt (c-PTIO), and the NO donors S-nitroso-N-acetylpenicillamine (SNAP) for more short-term NO effects (t_½ _≈ 50 min; dispersal experiment) or 3,3-Bis(aminoethyl)-1-1-hydroxy-2-oxo-1-triazene (Noc-18) in longer experiments (t_½ _≈ 3400 min; swarming and biofilm formation experiments). The theoretically expected time courses of NO release by the donors without concurrent loss processes in different experiments are shown in the additional file 1 (figures s1 and s2).

### Construction of *nos *knock-out

Deletion of *nos *gene from *B.subtilis *PY79 genome was achieved by long-flanking homology polymerase chain reaction (LFH-PCR) technique [37]. The deletion/insertion *nos::mls *was constructed by PCR amplifying approximately 1 kbp from 5'-flanking region of *nos *gene with primers P1b_BsNOS (5' taa cgg cat aca aca ttc cgg agg 3') and P2b_BsNOS (5' att atg tct ttt gcg cag tcg gcc ttt ttc ttc caa caa act ctc ccc 3'), while another band of near 1 kbp from 3'-flanking region was amplified using P3_BsNOS (5' cat tca att ttg agg gtt gcc agc aat cgt taa gcc gaa cta ttt tta tc 3') and P4_BsNOS (5' cgc gaa ctg gac gga tat gcc tt 3'). The resulting PCR products were then used as primers to amplify the erythromycin-resistance cassette from the plasmid pDG646 [38] as previously described [37]. This creates a deletion of the *nos *gene from nucleotide +12 to +1064 assuming the +1 nucleotide described in Adak *et al. *[5]. The PCR products were then transformed into PY79 as previously described [39] and the mutants were confirmed by PCR. The *nos::mls *mutation were then introduced in 3610 strain by SPP1 phage transduction [40,41] and confirmed by PCR analysis.

### Detection of intracellular NO formation

One milliliter overnight culture was inoculated in 50 mL LB and in 50 mL LB supplemented with 100 μM NOS inhibitor L-NAME. The culture was grown to the mid-exponential phase and was mixed with the NO sensitive dye CuFL (prepared according to suppliers instruction; Strem Chemicals, Newburyport, MA) [42] to reach a final concentration of 10 μM. In addition, cells grown to the mid-exponential phase in LB without L-NAME were mixed with NO scavenger c-PTIO to a final concentration of 100 μM and incubated for 1.5 h at room temperature prior to CuFL staining. Cells were incubated with CuFL for ~30 min, placed on microscopic glass slides and covered with poly-L-Lysine coated cover slips. NO imaging was performed with a Confocal Laser Scanning Microscope (LSM 510, Zeiss, Germany) equipped with a Plan-Apochromat 100×, NA 1.4 oil lens. CuFL was excited at a wavelength of 488 nm with an Argon ion laser. The beamsplitter in front of the laser was HFT 488/543. The detector was equipped with a bandpass filter BP 505-530. In a second scanning cycle transmission images were collected at a wavelength of 633 nm with the in-built photo-diode detector. Digital image processing was done with ImageJ software (National Institute of Health, Bethesda, MD). For quantification of relative fluorescence (representing NO concentrations) images were filtered by a 2 pixel wide gaussian kernel. The maximum fluorescence values of single cells were measured and corrected for the cell ambient background.

### Biofilm formation

The influence of NOS-derived NO on biofilm formation was tested by investigating the morphology and fine structure of spot colonies grown on MSgg fortified with 1.5% agar. Additionally, the amount of vegetative cells and spores in biofilms grown on the liquid-air interface ('pellicles') in MSgg medium was quantified. Both agar and medium were supplemented with sterile filtered (0.2 μm, Spartan, Millipore, Schwalbach, Germany) 100 μM L-NAME, 75 μM c-PTIO or 130 μM Noc-18 after autoclavation.

Colony morphology was investigated in 6-well microtiter plates (Nunclon Surface, Nunc, Denmark) and colony fine structure was investigated in Petri dishes (Sarstedt, Nümbrecht, Germany). The wells of the microtiter plates were filled with 6 mL and the Petri dishes with 25 mL MSgg agar. After the agar dried for ~ 16 h at room temperature (RT), 5 μL of a LB-grown overnight culture was spotted on the agar surface, dried open for 10 min in a laminar flow hood, and incubated at 26°C. Fine structure of 3 days old colonies was visualized by illuminating the sample with an external light source (swan neck lamp, KL 1500 electronic, Schott, Mainz, Germany) and capturing reflected light with a DS-Q1-MC CCD camera (Nikon, Japan) mounted on a light microscope (DM RA2, Leica, Solms, Germany) equipped with Leica 5× NA0.15 HC PL Fluotar lens. Whole colony morphology was documented with a digital camera after 4 days of growth.

Pellicle formation was quantified in glass test tubes containing 25 mL MSgg medium. MSgg tubes were inoculated with 25 μL of mid-exponential phase culture and incubated for 7 days at 26°C without agitation. Directly after the inoculation 980 μL medium was removed from the tube and subjected to NO staining with CuFL as described above. During the course of biofilm formation 3 vials of each treatment per day were sacrificed for determination of viable cell and spore counts. Biofilms were homogenized in the MSgg medium by sonication (Labsonic U, B. Braun, Melsungen, Germany) for 10 min at ~ 40 W on ice. The cells were plated on LB agar, and incubated 24 h at 26°C to determine the number of colony forming units (cfu). Spore counts were determined from the same samples by subjecting a part of the homogenates to pasteurization for 20 min at 80°C in a water bath prior to plating. O_2 _and NO concentrations in the biofilm incubations were measured with microsensors as previously described [43,44].

### Swarm expansion assay

Swarm experiments were conducted as described by Kearns and Losick [13]. Briefly, cells grown in LB at 37°C to the mid-exponential phase were harvested by centrifugation (15 min, 4000 RCF, 15°C) and re-suspended in phosphate buffered saline (137 mM NaCl, 2.7 mM KCl, 10 mM Na_2_HPO_4_, and 2 mM KH_2_PO_4_) containing 0.5% ink. Swarm plates were prepared in Petri dishes (diameter = 8.5 cm) by pouring 25 mL LB fortified with 0.7% agar and supplemented with 100 μM L-NAME, 100 μM c-PTIO or 20 μM and 200 μM Noc-18. The plates were dried for 30 min under a laminar flow hood, directly afterward inoculated with 3 × 10^8 ^cells within 10 μL in the centre of the plate, dried for another 10 min, and incubated at 37°C. The swarm radii were measured relative to the origin of swarming, which was demarked by the edge of the ink stained agar in the centre.

We used statistics to confirm that the differences between treatments were not significant. Normality of the data was confirmed with Saphiro-Wilk W test (α = 0.01). Comparison between different experimental treatments was performed by a One-Way-Analysis of Variance (α = 0.01) with NCSS software (PASS2000, Kaysville, UT). Turkey-Kramer post-hoc test was used to determine significant differences between individual factors.

### Dispersal assay

Spot colony biofilms were grown on agar in 6-well plates filled with MSgg agar, MSgg agar + 100 μM L-NAME and MSgg agar + 75 μM c-PTIO. After 4 days of growth a 100 μL drop MSgg medium was mounted on the colonies and incubated for 2 h at RT. The drops of the experimental treatments contained 100 μM L-NAME for MSgg/L-NAME agar, 750 μM c-PTIO for MSgg/c-PTIO agar, 300 μM SNAP for MSgg agar, and 100 μM L-NAME + 300 μM SNAP for MSgg/L-NAME agar. Next, 80 μL of the drop liquid were removed. The cells were fixed with formaldehyde at a final concentration of 3.7% and incubated at 4°C overnight. Cell counting was done with a flow cytometer (FACS Calibur, Becton Dickinson, Franklin Lakes, NJ) on the following day. The fixed cells were mixed with 500 μL sterile filtered, deionised water that contained fluorescent latex beads (AlignFlow, alignment beads 2.5 μm, Molecular Probes, Eugene, OR) and with 1×Cybr Green DNA stain (Molecular Probes, Eugene, OR). Vegetative cells were differentiated from spores based on their size difference. Cell counts per volume could be calculated based on the number of beads counted in each run and an initial calibration of the bead solution.

### Germination assay

MSgg medium was supplemented with the same treatments as used during the dispersal assay. Spores were prepared by growing *B. subtilis *in Difco sporulation medium (DSM) at 37°C for 16 h. After that time all cells in DSM were spores as determined by comparing direct plate counts to heat inactivated (80°C, 20 min) plate counts. Spores were added to MSgg and MSgg plus treatments to reach a final concentration of ~10^6 ^spores mL^-1^. 100 μL drops of the MSgg-spore suspensions were placed on sterile Petri dish surfaces and incubated for 2 h at RT. 80 μL of each drop were harvested and split in two parts: 40 μL were plated immediately on LB agar to determine the total cfu (vegetative cells + spores), while the other 40 μL were heated at 80°C for 20 min prior to LB-plating to determine the spore forming units.

### Microsensor measurements

NO microprofiles were measured in the same set-up as used in the dispersal assay. Spot colony biofilms were grown on MSgg agar in Petri dishes for 4 d. A 100 μL drop of MSgg was mounted on top of the biofilm and NO microprofiles were measured immediately with an NO microsensor as described previously [43]. For each experimental treatment, MSgg was supplied either with or without 300 μM of the NO donor SNAP. SNAP was mixed to MSgg directly before the experiment. Experimental treatments were as followed: (i) wild-type: *B. subtilis *3610 for which MSgg agar and drop were added without further supplementation; (ii) wild-type: *B. subtilis *3610 for which MSgg agar and drop were supplemented with 100 μM L-NAME; and (iii) *B. subtilis *3610 Δ*nos *for which MSgg agar and drop were added without further supplementation.

## Authors' contributions

FS and DdB conceived the research and analyzed the data. FS designed and performed the experiments, and drafted the manuscript. MB and FS performed NO imaging, quantified intracellular NO concentrations and imaged fruiting bodies. DE and FS designed and performed experiments on biofilm formation. MLG, OZ and JEGP constructed the *nos *knock-out mutant, performed the germination assay and contributed in experimental design and analysis. All Authors contributed in writing the manuscript and approved its final content.

## Supplementary Material

Additional file 1 **Figure S1. Theoretical formation of NO from the NO donor Noc-18**. The figure shows the calculated formation of NO over time for different starting concentrations of Noc-18. **Figure S2. Theoretical formation of NO from the NO donor SNAP.** The figure shows the calculated formation of NO over time for different starting concentrations of SNAP. Click here for file

## References

[B1] Bredt DS Snyder SH Nitric-Oxide - a Physiological Messenger Molecule Annu Rev Biochem 1994 63 175 195 10.1146/annurev.bi.63.070194.001135 7526779

[B2] Alderton WK Cooper CE Knowles RG Nitric oxide synthases: structure, function and inhibition Biochem J 2001 357 593 615 10.1042/0264-6021:3570593 11463332 PMC1221991

[B3] Stamler JS Lamas S Fang FC Nitrosylation: The prototypic redox-based signaling mechanism Cell 2001 106 675 683 10.1016/S0092-8674(01)00495-0 11572774

[B4] Sudhamsu J Crane BR Bacterial nitric oxide synthases: what are they good for? Trends Microbiol 2009 17 212 218 10.1016/j.tim.2009.02.003 19375324

[B5] Adak S Aulak KS Stuehr DJ Direct evidence for nitric oxide production by a nitric-oxide synthase-like protein from Bacillus subtilis J Biol Chem 2002 277 16167 16171 10.1074/jbc.M201136200 11856757

[B6] Gusarov I Nudler E NO-mediated cytoprotection: Instant adaptation to oxidative stress in bacteria Proc Natl Acad Sci USA 2005 102 13855 13860 10.1073/pnas.0504307102 16172391 PMC1236549

[B7] Gusarov I Shatalin K Starodubtseva M Nudler E Endogenous Nitric Oxide Protects Bacteria Against a Wide Spectrum of Antibiotics Science 2009 325 1380 1384 10.1126/science.1175439 19745150 PMC2929644

[B8] Kers JA Wach MJ Krasnoff SB Widom J Cameron KD Bukhalid RA Gibson DM Crane BR Loria R Nitration of a peptide phytotoxin by bacterial nitric oxide synthase Nature 2004 429 79 82 10.1038/nature02504 15129284

[B9] Spiro S Regulators of bacterial responses to nitric oxide Fems Microbiol Rev 2007 31 193 211 10.1111/j.1574-6976.2006.00061.x 17313521

[B10] Zumft WG Nitric oxide reductases of prokaryotes with emphasis on the respiratory, heme-copper oxidase type J Inorg Biochem 2005 99 194 215 10.1016/j.jinorgbio.2004.09.024 15598502

[B11] Aguilar C Vlamakis H Losick R Kolter R Thinking about Bacillus subtilis as a multicellular organism Curr Opin Microbiol 2007 10 638 643 10.1016/j.mib.2007.09.006 17977783 PMC2174258

[B12] Branda SS Gonzalez-Pastor JE Ben-Yehuda S Losick R Kolter R Fruiting body formation by Bacillus subtilis Proc Natl Acad Sci USA 2001 98 11621 11626 10.1073/pnas.191384198 11572999 PMC58779

[B13] Kearns DB Losick R Swarming motility in undomesticated Bacillus subtilis Mol Microbiol 2003 49 581 590 12864845 10.1046/j.1365-2958.2003.03584.x

[B14] Lopez D Kolter R Extracellular signals that define distinct and coexisting cell fates in Bacillus subtilis Fems Microbiol Rev 2010 34 134 149 10.1111/j.1574-6976.2009.00199.x 20030732

[B15] Gonzalez-Pastor JE Graumann P Multicellularity and social behaviour in Bacillus subtilis Bacillus: Cellular and Molecular Biology 2007 Wymondham, UK: Horizon Scientific Press-Caister Academic Press 149 419

[B16] Zeigler DR Pragai Z Rodriguez S Chevreux B Muffler A Albert T Bai R Wyss M Perkins JB The Origins of 168, W23, and Other Bacillus subtilis Legacy Strains J Bacteriol 2008 190 6983 6995 10.1128/JB.00722-08 18723616 PMC2580678

[B17] Earl AM Losick R Kolter R Bacillus subtilis genome diversity J Bacteriol 2007 189 1163 1170 10.1128/JB.01343-06 17114265 PMC1797320

[B18] Fraser GM Hughes C Swarming motility Curr Opin Microbiol 1999 2 630 635 10.1016/S1369-5274(99)00033-8 10607626

[B19] Karatan E Watnick P Signals, Regulatory Networks, and Materials That Build and Break Bacterial Biofilms Microbiol Mol Biol Rev 2009 73 310 + 10.1128/MMBR.00041-08 19487730 PMC2698413

[B20] Sauer K Camper AK Ehrlich GD Costerton JW Davies DG Pseudomonas aeruginosa displays multiple phenotypes during development as a biofilm J Bacteriol 2002 184 1140 1154 10.1128/jb.184.4.1140-1154.2002 11807075 PMC134825

[B21] Purevdorj-Gage B Costerton WJ Stoodley P Phenotypic differentiation and seeding dispersal in non-mucoid and mucoid Pseudomonas aeruginosa biofilms Microbiology-(UK) 2005 151 1569 1576 10.1099/mic.0.27536-0 15870466

[B22] Gjermansen M Ragas P Sternberg C Molin S Tolker-Nielsen T Characterization of starvation-induced dispersion in Pseudomonas putida biofilms Environ Microbiol 2005 7 894 906 10.1111/j.1462-2920.2005.00775.x 15892708

[B23] Hunt SM Werner EM Huang BC Hamilton MA Stewart PS Hypothesis for the role of nutrient starvation in biofilm detachment Appl Environ Microbiol 2004 70 7418 7425 10.1128/AEM.70.12.7418-7425.2004 15574944 PMC535154

[B24] Sauer K Cullen MC Rickard AH Zeef LAH Davies DG Gilbert P Characterization of nutrient-induced dispersion in Pseudomonas aeruginosa PAO1 biofilm J Bacteriol 2004 186 7312 7326 10.1128/JB.186.21.7312-7326.2004 15489443 PMC523207

[B25] Nakano MM Zuber P Anaerobic growth of a "strict aerobe" (Bacillus subtilis) Annu Rev Microbiol 1998 52 165 190 10.1146/annurev.micro.52.1.165 9891797

[B26] Gusarov I Starodubtseva M Wang ZQ McQuade L Lippard SJ Stuehr DJ Nudler E Bacterial nitric-oxide Synthases operate without a dedicated redox partner J Biol Chem 2008 283 13140 13147 10.1074/jbc.M710178200 18316370 PMC2442334

[B27] Corker H Poole RK Nitric oxide formation by Escherichia coli - Dependence on nitrite reductase, the NO-sensing regulator FNR, and flavohemoglobin Hmp J Biol Chem 2003 278 31584 31592 10.1074/jbc.M303282200 12783887

[B28] Baruah A Lindsey B Zhu Y Nakano MM Mutational analysis of the signal-sensing domain of ResE histidine kinase from Bacillus subtilis J Bacteriol 2004 186 1694 1704 10.1128/JB.186.6.1694-1704.2004 14996800 PMC355969

[B29] Kolodkin-Gal I Romero D Cao SG Clardy J Kolter R Losick R D-Amino Acids Trigger Biofilm Disassembly Science 2010 328 627 629 10.1126/science.1188628 20431016 PMC2921573

[B30] Barraud N Hassett DJ Hwang SH Rice SA Kjelleberg S Webb JS Involvement of nitric oxide in biofilm dispersal of Pseudomonas aeruginosa J Bacteriol 2006 188 7344 7353 10.1128/JB.00779-06 17050922 PMC1636254

[B31] Barraud N Schleheck D Klebensberger J Webb JS Hassett DJ Rice SA Kjelleberg S Nitric Oxide Signaling in Pseudomonas aeruginosa Biofilms Mediates Phosphodiesterase Activity, Decreased Cyclic Di-GMP Levels, and Enhanced Dispersal J Bacteriol 2009 191 7333 7342 10.1128/JB.00975-09 19801410 PMC2786556

[B32] Barraud N Storey MV Moore ZP Webb JS Rice SA Kjelleberg S Nitric oxide-mediated dispersal in single- and multi-species biofilms of clinically and industrially relevant microorganisms Microbial Biotechnology 2009 2 370 378 10.1111/j.1751-7915.2009.00098.x 21261931 PMC3815757

[B33] Zumft WG Nitric oxide signaling and NO dependent transcriptional control in bacterial denitrification by members of the FNR-CRP regulator family J Mol Microbiol Biotechnol 2002 4 277 286 11931559

[B34] Firoved AM Wood SR Ornatowski W Deretic V Timmins GS Microarray analysis and functional characterization of the nitrosative stress response in nonmucoid and mucoid Pseudomonas aeruginosa J Bacteriol 2004 186 4046 4050 10.1128/JB.186.12.4046-4050.2004 15175322 PMC419947

[B35] Nakano MM Induction of ResDE-dependent gene expression in Bacillus subtilis in response to nitric oxide and nitrosative stress J Bacteriol 2002 184 1783 1787 10.1128/JB.184.6.1783-1787.2002 11872732 PMC134876

[B36] Moore CM Nakano MM Wang T Ye RW Helmann JD Response of Bacillus subtilis to nitric oxide and the nitrosating agent sodium nitroprusside J Bacteriol 2004 186 4655 4664 10.1128/JB.186.14.4655-4664.2004 15231799 PMC438601

[B37] Wach A PCR-synthesis of marker cassettes with long flanking homology regions for gene disruptions in S-cerevisiae Yeast 1996 12 259 265 10.1002/(SICI)1097-0061(19960315)12:3<259::AID-YEA901>3.0.CO;2-C 8904338

[B38] GueroutFleury AM Shazand K Frandsen N Stragier P Antibiotic-resistance cassettes for Bacillus subtilis Gene 1995 167 335 336 10.1016/0378-1119(95)00652-4 8566804

[B39] Spizizen J Transformation of Biochemically Deficient Strains of Bacillus-Subtilis by Deoxyribonucleate Proc Natl Acad Sci USA 1958 44 1072 1078 10.1073/pnas.44.10.1072 16590310 PMC528696

[B40] Yasbin RE Young FE Transduction in Bacillus-Subtilis by Bacteriophage Spp1 J Virol 1974 14 1343 1348 4214946 10.1128/jvi.14.6.1343-1348.1974 PMC355660

[B41] Kearns DB Chu F Rudner R Losick R Genes governing swarming in Bacillus subtilis and evidence for a phase variation mechanism controlling surface motility Mol Microbiol 2004 52 357 369 10.1111/j.1365-2958.2004.03996.x 15066026

[B42] Lim MH Xu D Lippard SJ Visualization of nitric oxide in living cells by a copper-based fluorescent probe Nat Chem Biol 2006 2 375 380 16732295 10.1038/nchembio794

[B43] Schreiber F Polerecky L de Beer D Nitric oxide microsensor for high spatial resolution measurements in biofilms and sediments Anal Chem 2008 80 1152 1158 10.1021/ac071563x 18197634

[B44] Revsbech NP An Oxygen Microsensor with a Guard Cathode Limnol Oceanogr 1989 34 474 478 10.4319/lo.1989.34.2.0474

