# Supplementary material for: The role of nitric-oxide-synthase-derived nitric oxide in multicellular traits of Bacillus subtilis 3610: biofilm formation, swarming, and dispersal
Source: BMC Microbiol. 2011 May 20;11:111. doi: 10.1186/1471-2180-11-111 (PMC3224222; doi:10.1186/1471-2180-11-111)
Supplement: Additional file 1 — Figure S1. Theoretical formation of NO from the NO donor Noc-18. The figure shows the calculated formation of NO over time for different starting concentrations of Noc-18. Figure S2. Theoretical formation of NO from the NO donor SNAP. The figure shows the calculated formation of NO over time for different starting concentrations of SNAP. [file 1471-2180-11-111-S1.PDF]

Supplementary Information

BMC Microbiology

The Role of Nitric-oxide-synthase-derived Nitric Oxide in multicellular traits of Bacillus subtilis 3610: Biofilm Formation, Swarming, and Dispersal

Frank Schreiber[a*], Martin Beutler[a,b], Dennis Enning[a], María Lamprecht-Grandio[c], Olga Zafra[c], José Eduardo González-Pastor[c], and Dirk de Beer[a]

[a]*Max-Planck-Institute for Marine Microbiology, Celsiusstrasse 1, D-28359 Bremen, Germany*
[b]*bionsys GmbH, Fahrenheitstrasse 1, D-28359, Bremen, Germany*
[c]*Instituto Nacional de Téchnica Aeroespecial, Centro de Astrobiologia (INTA-CSIC), Madrid 28850, Spain*

[*] corresponding author: Frank Schreiber

e-mail: frank.schreiber@eawag.ch

present address: *Eawag - Swiss Federal Institute of Aquatic Science and Technology, Überlandstrasse 133 P.O. Box 611 8600 Dübendorf Switzerland*

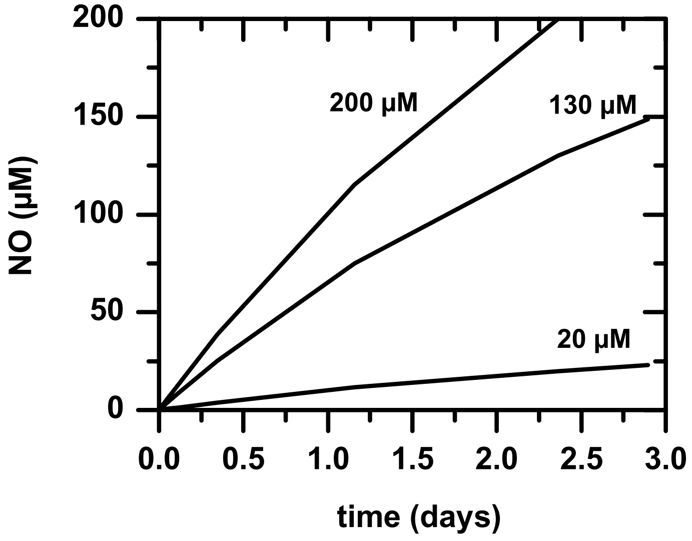

Fig. S1. Theoretical formation of NO from the NO donor Noc-18 based on a half-life of 3400 min (Calbiochem Data Sheet 487957). Different starting concentrations of Noc-18 are indicated on the graph. 20 µM and 200 µM Noc-18 were used in swarming experiments which were conducted within approx. 1 day. 130 µM Noc-18 were used in biofilm experiments with the major activity within the first 3 days of the experiment.

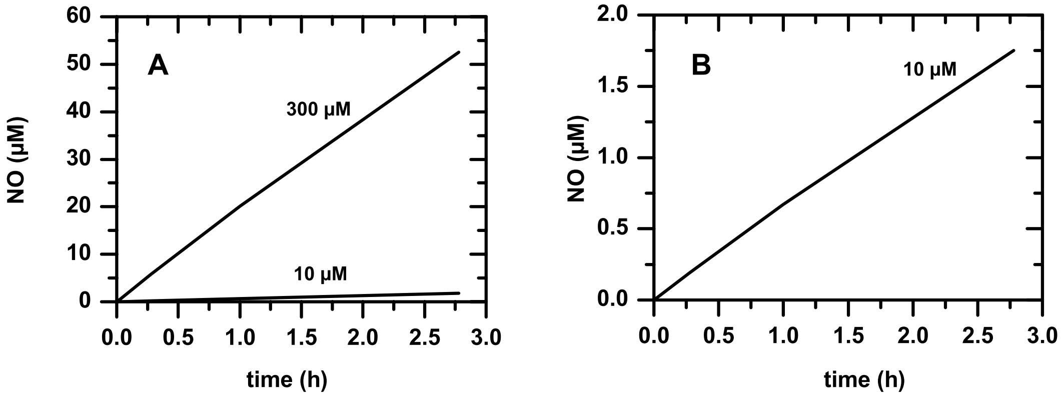

Fig. S2. Theoretical formation of NO from the NO donor SNAP based on a half-life of 600 min (Calbiochem Data Sheet 487910). Panel **(A)** shows starting concentrations of 10 µM and 300 µM SNAP as used in the dispersal experiments, which was conducted within 2 hours. Panel **(B)** shows in amplification of panel A with different y-scaling.
